# Robustness and Generalizability of Deep Learning Synthetic Computed Tomography for Positron Emission Tomography/Magnetic Resonance Imaging–Based Radiation Therapy Planning of Patients With Head and Neck Cancer

**DOI:** 10.1016/j.adro.2021.100762

**Published:** 2021-07-26

**Authors:** Anders B. Olin, Christopher Thomas, Adam E. Hansen, Jacob H. Rasmussen, Georgios Krokos, Teresa Guerrero Urbano, Andriana Michaelidou, Björn Jakoby, Claes N. Ladefoged, Anne K. Berthelsen, Katrin Håkansson, Ivan R. Vogelius, Lena Specht, Sally F. Barrington, Flemming L. Andersen, Barbara M. Fischer

**Affiliations:** a Department of Clinical Physiology, Nuclear Medicine and PET, Rigshospitalet, University of Copenhagen, Copenhagen, Denmark; b Department of Medical Physics, Guy's and St Thomas' NHS Foundation Trust, London, United Kingdom; c Faculty of Health and Medical Science, University of Copenhagen, Copenhagen, Denmark; d Department of Radiology, Rigshospitalet, University of Copenhagen, Copenhagen, Denmark; e Department of Otorhinolaryngology, Head & Neck Surgery and Audiology, Rigshospitalet, University of Copenhagen, Copenhagen, Denmark; f Department of Otorhinolaryngology and Maxillofacial Surgery, Zealand University Hospital, Køge, Denmark; g King's College London and Guy's and St Thomas’ PET Centre, School of Biomedical Engineering and Imaging Sciences, King's College London, King's Health Partners, London, United Kingdom; h Department of Oncology, Guy's and St Thomas’ NHS Foundation Trust, London, United Kingdom; i Siemens Healthcare GmbH, Erlangen, Germany; j University of Surrey, Guildford, Surrey, United Kingdom; k Department of Oncology, Section of Radiotherapy, Rigshospitalet, University of Copenhagen, Copenhagen, Denmark

## Abstract

**Purpose:**

Radiotherapy planning based only on positron emission tomography/magnetic resonance imaging (PET/MRI) lacks computed tomography (CT) information required for dose calculations. In this study, a previously developed deep learning model for creating synthetic CT (sCT) from MRI in patients with head and neck cancer was evaluated in 2 scenarios: (1) using an independent external dataset, and (2) using a local dataset after an update of the model related to scanner software-induced changes to the input MRI.

**Methods and Materials:**

Six patients from an external site and 17 patients from a local cohort were analyzed separately. Each patient underwent a CT and a PET/MRI with a Dixon MRI sequence over either one (external) or 2 (local) bed positions. For the external cohort, a previously developed deep learning model for deriving sCT from Dixon MRI was directly applied. For the local cohort, we adapted the model for an upgraded MRI acquisition using transfer learning and evaluated it in a leave-one-out process. The sCT mean absolute error for each patient was assessed. Radiotherapy dose plans based on sCT and CT were compared by assessing relevant absorbed dose differences in target volumes and organs at risk.

**Results:**

The MAEs were 78 ± 13 HU and 76 ± 12 HU for the external and local cohort, respectively. For the external cohort, absorbed dose differences in target volumes were within ± 2.3% and within ± 1% in 95% of the cases. Differences in organs at risk were <2%. Similar results were obtained for the local cohort.

**Conclusions:**

We have demonstrated a robust performance of a deep learning model for deriving sCT from MRI when applied to an independent external dataset. We updated the model to accommodate a larger axial field of view and software-induced changes to the input MRI. In both scenarios dose calculations based on sCT were similar to those of CT suggesting a robust and reliable method.

## Introduction

The use of combined positron emission tomography (PET)/magnetic resonance imaging (MRI) offers new possibilities for individualized radiotherapy planning as it provides spatially and temporally aligned structural and functional information in a single examination.^1^ The excellent soft tissue contrast of MRI is ideal for target delineation and biological tumor characterization based on functional information from both PET and MRI for dose painting and adaptive planning strategies.^2^, ^3^, ^4^ The development of dedicated radiotherapy equipment, which is compatible with MRI and PET means it is possible to integrate PET/MRI in the radiotherapy workflow^5^^,^^6^ with the aim of completely replacing the routine planning CT to eliminate systematic registration errors between scan sessions^7^^,^^8^ and reduce scan time. Studies concerning head and neck cancer have demonstrated the feasibility of scanning patients with PET/MRI in the radiotherapy treatment position using dedicated equipment such as flat table overlay and immobilization masks.^9^^,^^10^

Information about the electron density of tissue is a prerequisite for dose calculation, which is provided by CT to a very good approximation, but not by MRI. Similar information is needed for PET attenuation correction (AC) in the context of PET/MRI. Consequently, several studies have reported methods for generating synthetic CT (sCT) from MRI with promising results, especially in the brain and the pelvic region using a variety of different approaches.^11^, ^12^, ^13^, ^14^, ^15^, ^16^ The number of studies in head and neck is more limited and while initial methods have used atlas-based approaches^14^^,^^17^^,^^18^ the challenging complex anatomy with large inter-patient variations and abnormal anatomy raise the need for an alternative strategy.

Recently, deep learning algorithms such as convolutional neural networks have been derived for the head and neck region demonstrating great results.^10^^,^^19^^,^^20^ Data used to train and test such networks are often selected from a well-characterized group of patients from a single site and a single scanner resulting in a model, which is tuned to specific training data. In a clinical setup, robustness and generalizability are critical for methods to be successfully applied across sites and scanners. Deep learning methods developed locally must therefore be tested in external independent test data. In scenarios where input data significantly differ from the data originally used for training (eg, due to a permanent change in acquisition protocol), it might be necessary to update the model through transfer learning. This is a highly regarded strategy to update/fine-tune a model, allowing for a significant reduction in training data.^21^

In this study, we aimed to evaluate the robustness and generalizability of a previously developed deep learning model for creating sCT from MRI in head and neck patients. We evaluated the model performance for radiotherapy when: (1) applied to an independent external dataset from another site, and (2) the model was updated to accommodate input MRI with a larger axial field of view (FOV) and changes in MRI sequence parameters induced by a scanner software upgrade.

## Methods and Materials

Patient data from 2 sites were included in this study; 6 patients from an external site (Guy’s and St. Thomas’ Hospital, London, United Kingdom), and 17 patients from the local site (Rigshospitalet, University Hospital Copenhagen, Copenhagen, Denmark). All patients were referred for radiotherapy of head and neck cancer (except one gastrointestinal patient with upper esophageal cancer). All patients gave written informed consent and study participation did not alter the planned treatment at either site. The studies were approved by the local ethics committees.

### Imaging

All patients underwent either a planning CT (external site) or a planning [^18^F]FDG-PET/CT (local site) as part of the clinical routine of the individual site. CT scan parameters are specified in Table 1 and imaging was performed in the treatment position using flat table overlays and thermoplastic fixation masks for patient immobilization as per international standard. Subsequently, [^18^F]FDG-PET/MRI examination was performed in the same treatment position using the same fixation mask as for the preceding planning CT or PET/CT examination.^5^^,^^10^^,^^22^ Images from the 2 scan sessions were coregistered by nonrigid alignment (reg_f3d, NiftyReg)^23^ after an initial rigid registration (reg_aladin, NiftyReg).

**Table 1:** Technical details concerning imaging and dose planning at the 2 sites

	Dataset	
	External (n = 6)	Local (n = 17)
Planning CT		
Scanner	SOMATOM definition AS (CT)	Biograph TruePoint 64 (PET/CT)
Examination type	Whole body	Whole body
X-ray tube voltage	120 kVp	100 kVp/120 kVp
CT intravenous contrast	Yes	Yes
Reconstruction matrices	512 × 512	512 × 512
Pixel spacing, mm^2^	0.98 × 0.98	1.52 × 1.52
Slice thickness, mm	2	2
PET/MRI		
Scanner	Biograph mMR (PET/MRI)	Biograph mMR (PET/MRI)
Software version	VB20P (old)	VE11P (new)
Examination type	Regional	Regional
Dixon AC sequence		
TR/TE1/TE2, ms	3.60/1.23/2.46	3.85/1.23/2.46
Orientation	Coronal (x,z)	Transaxial (x,y)
Reconstruction matrices	192 × 126 × 128 (x,z,y)	384 × 312 × 88 (x,y,z)
Pixel spacing, mm^2^	2.6 × 2.6 (x,z)	1.3 × 1.3 (x,y)
Slice thickness, mm	3.1 (y)	3.0 (z)
Bed-positions	1	2
MR-AC map with bone	No	Yes
Treatment planning		
TPS	Monaco	Eclipse
Delivery technique	VMAT (2 arcs)	VMAT (2 arcs)
Prescribed dose, Gy	65	68 (66 post surgery)
Dose calculation		
Calculation model	Monte Carlo (0.3% statistical uncertainty per plan)	AcurosXB
Reported dose	Dose to medium	Dose to medium
Grid spacing, mm^2^	2.5 × 2.5	2.5 × 2.5
Grid thickness, mm	2.5	3.0

*Abbreviations*: CT = computed tomography; MR-AC = magnetic resonance-based attenuation correction; MRI = magnetic resonance imaging; PET = positron emission tomography; TPS = XXX; VMAT = volumetrically modulated arc therapy.

Relevant details about the PET/MRI examinations are given in Table 1. PET/MRI was performed on the same scanner model (Siemens Biograph mMR) but with different software versions across the 2 sites. At both sites, the MRI protocol included the vendor-provided Dixon sequence, which was performed over either one (external cohort) or 2 (local cohort) bed positions. This sequence produces 2 image volumes where signals from water and fat are in-phase and opposed-phase, respectively. However, as the vendor has pursued Dixon images of diagnostic quality, certain parameters were changed after upgrading the software versions (Table 1). In particular, the newer software version (VE11P) achieves a higher resolution while covering approximately the same FOV in the same scan time (19 seconds) as the older version (VB20P) due to CAIPIRINHA (controlled aliasing in volumetric parallel imaging) acceleration.^24^

The scanner uses the Dixon sequence to perform MR-based attenuation correction (MR-AC) of PET. The scanner derives an MR-AC map, which is a segmentation of the Dixon images of the patient into different tissue classes: soft tissue, fat, lung, and air each with a fixed linear attenuation coefficient value.^25^ After the software upgrade, the vendor-provided MR-AC map includes major bones from the skull and spine, which are superimposed onto the segmented Dixon images (MR-AC_Bone_).^26^

### sCT generation

The method for generating sCT was a deep convolutional neural network with a 3-dimensional (3D) U-net architecture, which was presented previously (https://github.com/andersolin/DeepMRAC_headneck).^10^ The model was originally trained on voxel-to-voxel matched pairs of CT (converted into LAC values) and Dixon MRI of the head (n = 811) and fine-tuned to the head and neck region (n = 11). The MRI data were acquired at the local site on a Siemens Biograph mMR prior to the software upgrade (ie, VB20P). The model takes 16 full adjacent axial slices from each of the Dixon in-phase and opposed-phase MRIs as a 2-channel input and yields the corresponding slices of a sCT given in LAC values. Preprocessing of input images entails resampling to isotropic voxels (2.04 × 2.04 × 2.04 mm^3^) in 240 × 192 matrices before normalizing to zero mean and unit standard deviation. A full sCT volume can be generated by predicting 16 full axial slices in a slice-by-slice manner throughout the MRI volume and composing the outputs into one volume by averaging overlapping slices. We refer to this model as the original model. This original model was directly applied to the external patient cohort to test the cross-site robustness.

For the local cohort, we created an updated model using transfer learning from the original model to accommodate the extended axial FOV and exploit the improved MRI resolution after the software upgrade. The training and evaluation in this step was performed in a leave-one-out process using the local dataset. Prior to training the model, contrast artifacts in the reference CT were manually set to the value of water and the 2 bed position MRIs were composed into one volume by normalizing the individual volumes to their combined global average before averaging overlapping slices (mincaverage; McConnel Imaging Center). The composed MRIs and the reference CT were preprocessed as in Olin et al^10^ but with image resampling into a smaller isotropic voxel size (1.3 × 1.3 × 1.3 mm^3^) in 416 × 288 matrices. To keep the subsequent validation as close to an independent test as possible, both the model architecture and training parameters were kept similar to the original model except for the loss function, which was changed to mean absolute error, as this is known to be less noise sensitive and causes less blurring.^27^^,^^28^ Training was performed with batch size of 12 in 20 steps per epochs for a total of 200 epochs on an IBM POWER9 server with 4 NVIDIA TESLA V100 GPUs.

All of the derived sCT images and the MR-AC_Bone_ maps were given in linear attenuation coefficients at 511 keV and converted to HU according to a bilinear scaling assuming an x-ray tube voltage corresponding to that of the reference CT.^29^ Finally, all sCT images and MR-AC_Bone_ maps were resampled to match the resolution of the reference CT using a trilinear interpolation (mincresample; McConnel Imaging Center).

### sCT evaluation

Each sCT was evaluated by comparing directly to the CT. For each patient the mean error (ME) and mean absolute error (MAE) between sCT and CT (sCT-CT) was calculated for the patient body, as well as air/lung (voxels below −200 HU in CT), bone (above 250 HU), and soft tissue compartments (between −200 and 250 HU). We also assessed the dice coefficients for bone and air/lungs. For the local cohort, these results were compared with those obtained using MR-AC_Bone_ maps. For this cohort, we further performed a visual one by one inspection of each sCT/CT pair and the corresponding Dixon MRI to identify regions where the sCT typically differs from the reference CT.

### Dosimetric evaluation

The effect of using sCT for calculating dose distributions was evaluated separately for the 2 sites. For each patient, a CT-based volumetrically modulated arc therapy treatment plan was created according to the local guidelines (see technical details in Table 1). Streaking artifacts caused by metal implants were manually delineated and the CT image value was overwritten with a HU value of 0. The optimized volumetrically modulated arc therapy plans, together with all delineated volumes, were copied onto the sCT and recalculated without modifications. No modifications were done to the sCT prior to dose calculation. The sCT-based and CT-based dose distributions were compared by gamma map (γ-map) analyses^30^ and dose-volume histogram (DVH) evaluations. For these analyses, we excluded all patients scanned with mouthpieces, as these are not visible on MRI together with patients with large MRI artifacts significantly affecting the sCT quality.

Local 3D gamma maps (γ-maps) with different acceptance criteria (eg, 2% difference between local doses within 2 mm [γ_2%/2mm_] were calculated for each sCT and pass rates (fraction of voxels passing the given criteria) were assessed within different planning target volumes: the primary (PTV1), the high risk of subclinical spread (PTV2), and the low risk of subclinical spread (PTV3). For the local cohort, γ-maps also were calculated using the MR-AC_Bone_ maps.

Using the DVHs of both the CT-based and sCT-based dose distributions, we assessed differences in relevant absorbed doses for PTV1, PTV2 and PTV3 as well as different organs at risk (OARs; ie, brain stem, spinal cord, and left/right parotid). The following absorbed doses were calculated for all regions of interest: the mean and maximum dose (D_mean_ and D_max_), the minimum dose given to 2%, 50%, 98%, 1 mL and 0.1 mL of the volume (D_2%_, D_50%_, D_98%_, D_1cc_, and D_0.1cc_).

## Results

### External cohort

**Figure 1:**
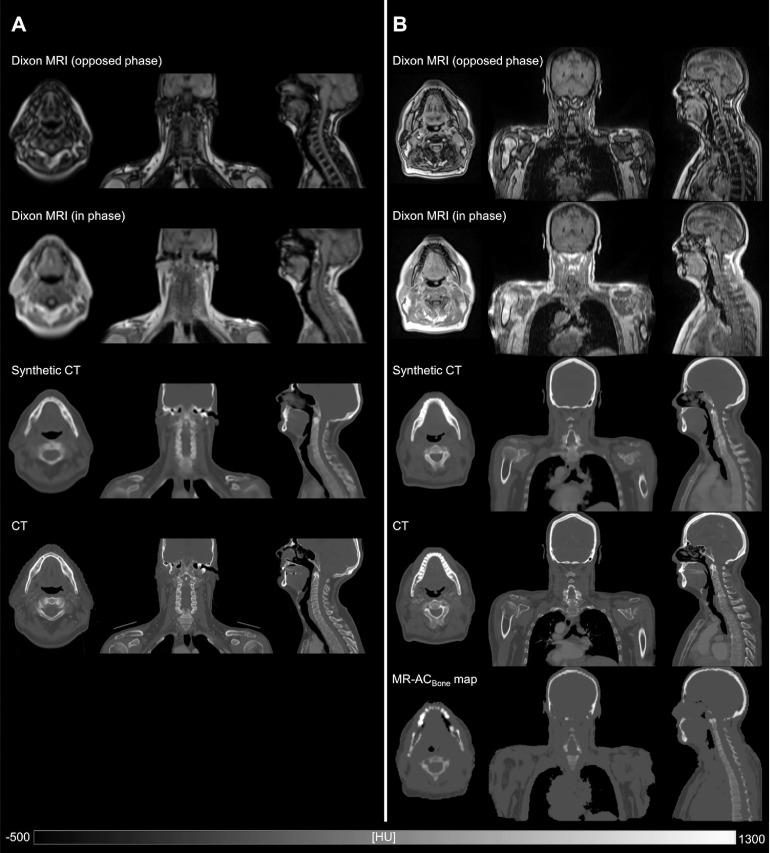
A patient example from the external cohort (A) and the local cohort (B). From top to bottom: The water and fat opposed-phase and in-phase Dixon magnetic resonance imaging (MRI), which serve as model input. The synthetic computed tomography derived from either the original model (A) or the updated model (B). The reference computed tomography. The MR-AC_Bone_ map (only in B). Notice the improved MRI resolution and the increased axial field-of-view for B compared with A.

**Figure 2:**
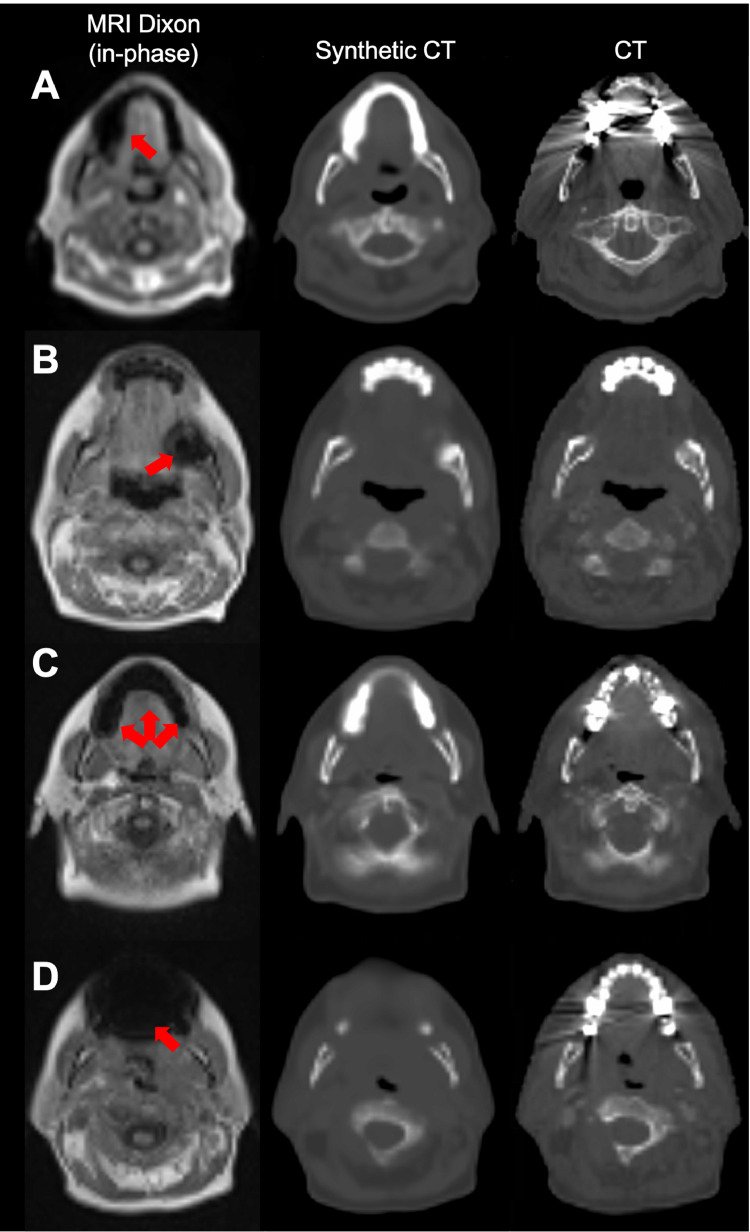
Cases illustrating the model's ability to handle metallic dental implants. (A) A case from the external cohort, where the dental implant caused severe streaking artifacts in the computed tomography (CT) and a signal void in the magnetic resonance imaging without translating significantly into the synthetic CT. (B-D) Cases from the local cohort, where dental implants only slightly affected the CT, but caused larger signal voids in the magnetic resonance images (MRI). For these cases the artifacts translated in varying degree into the synthetic CT images. Metal artifacts are marked on the MRI by red arrows.

Results of the quantitative comparison of sCT to CT in terms of ME, MAE, and the dice coefficients are shown in Table 2. The ME metrics show that soft tissue values are close to the reference CT, but for the entire body sCT values are underestimated, which is primarily driven by the underestimation of bone values. The sCT of a representative patient from the external cohort is shown in Figure 1A alongside the input MRIs and the corresponding reference CT. Visually, the sCT is similar to the reference CT but with a slightly blurred appearance. Figure 2A, shows a patient case where a dental implant causes streaking artifacts on the CT but has no severe impact on the MRI nor on the resulting sCT, which does not exhibit any significant artifacts.

**Table 2:** Quantitative evaluation of sCT with CT as reference and vendor-provided MR-AC_Bone_ maps for comparison

	Dataset		
	External (VB20P data)	Local (VE11P data)	
	sCT	MR-AC_Bone_ map	sCT
ME, HU			
Body	−22 ± 14	−43 ± 14	−14 ± 13
	(−52; −10)	(−66; −17)	(−43; 9)
Soft tissue	−9 ± 6	−18 ± 8	−3 ± 9
	(−21; −3)	(−31; −3)	(−21; 13)
Air/lungs	54 ± 18	95 ± 81	37 ± 44
	(27; 74)	(−46; 352)	(−77; 98)
Bone	−199 ± 60	−459 ± 42	−189 ± 44
	(−288; −120)	(−553; −378)	(−278; −101)
MAE, HU			
Body	78 ± 13	130 ± 10	76 ± 12
	(68; 105)	(114; 156)	(62; 120)
Soft tissue	48 ± 3	78 ± 7	48 ± 10
	(45; 54)	(65; 89)	(37; 83)
Air/lungs	117 ± 12	200 ± 73	121 ± 64
	(107; 142)	(137; 456)	(81; 239)
Bone	257 ± 45	500 ± 39	271 ± 33
	(192; 321)	(429; 592)	(224; 387)
Dice			
Bone	0.67 ± 0.03	0.38 ± 0.04	0.67 ± 0.05
	(0.62; 0.73)	(0.28; 0.45)	(0.58; 0.76)
Air/lungs	0.91 ± 0.01	0.80 ± 0.11	0.89 ± 0.05
	(0.89; 0.93)	(0.42; 0.91)	(0.72; 0.95)

The average mean error (ME) and mean absolute error (MAE) ( ± standard deviation and range) for different tissue compartments across all patients of each site. Average dice coefficient ± standard deviation and range for bone and air/lungs compartments.

*Abbreviations*: CT = computed tomography; MR-AC = magnetic resonance-based attenuation correction; sCT = synthetic computed tomography.

**Figure 3:**
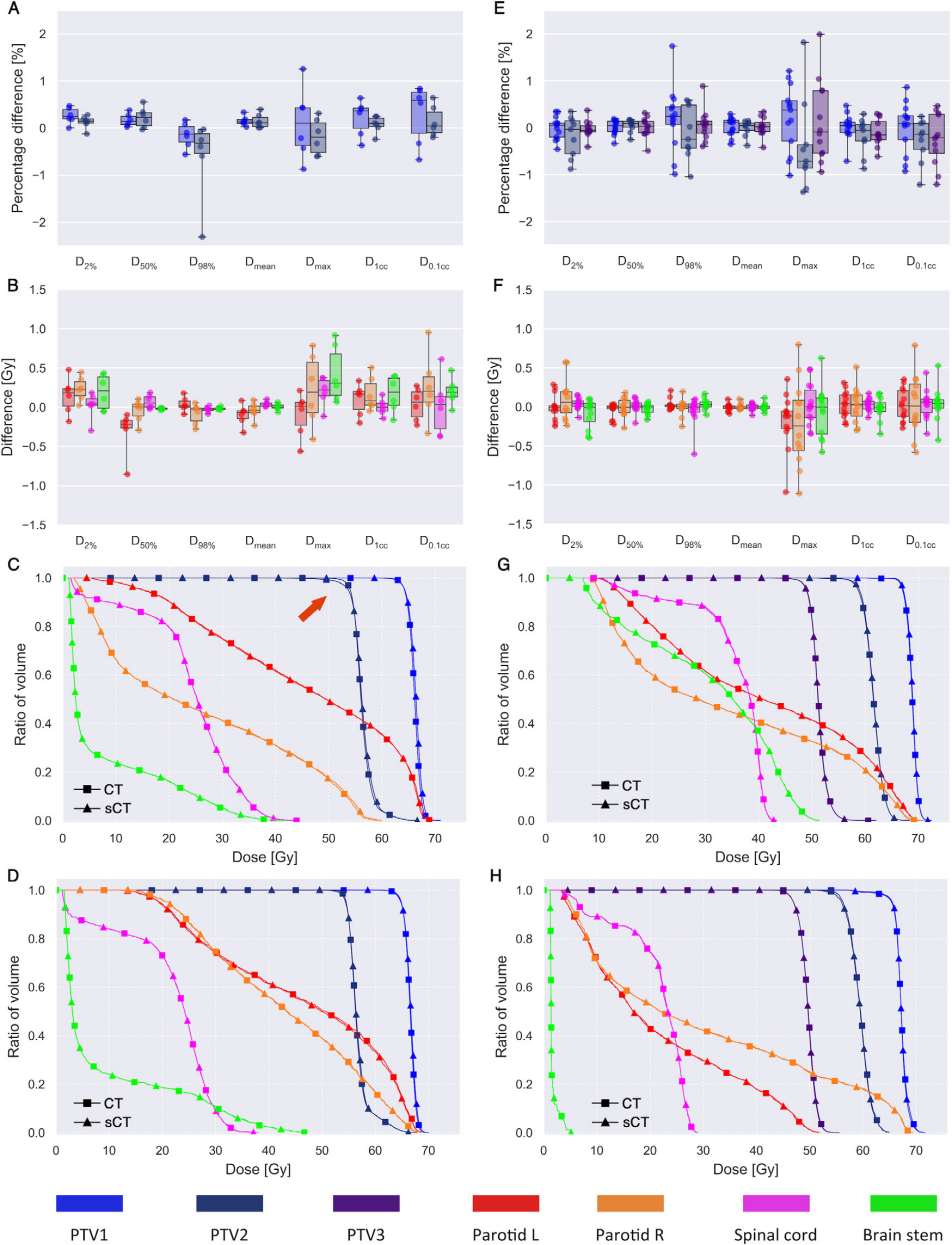
Dosimetric results for the external patient cohort (A, B, C, D) and the local patient cohort (E, F, G, H). (A, E) Scatter plots with box-whiskers (box shows the quartiles of the data; whiskers show the range of the data) of the relative dose difference between dose-volume histogram (DVH) points of the synthetic computed tomography-based dose distribution and the computed tomography-based dose distribution for the planning target volumes (PTV: primary [PTV1], the high risk of subclinical spread [PTV2], and the low risk of subclinical spread [PTV3], if available). (B, F) The dose difference between DVH points for the organs at risk (left/right parotid, spinal cord, and brainstem). (C, D, G, H) Patient examples of DVH curves. In (C) the red arrow indicates a −2.3% difference in D_98%_ for PTV2 (as seen in panel A).

Results of the gamma map analyses (Table 3) show a high agreement between the sCT-based and CT-based dose calculations with an average γ_2%/2mm_ pass rate of 98.9 ± 0.9% for PTV1. Differences in DVH points between the dose calculations are seen in Figure 3A and 3B. For PTV1 and PTV2 relative differences are less than ± 1.3% for all patients except one case (−2.3%/−1.2 Gy seen for D_98%_ of PTV2; Fig. 3A). The absolute differences in DVH points for all OARs are within ± 1 Gy, and most (95% of cases) within ± 0.5 Gy (Fig. 3B). The corresponding relative differences are <2% for all of the OARs, when including volumes with a CT-based D_max_ above 10 Gy. Figure 3C and 3D show the DVH curves for 2 patients of the external cohort, including the patient where the difference in D_98%_ of PTV2 was −2.3% (Fig. 3C).

**Table 3:** The average pass rate ( ± standard deviation and range) for gamma maps with 2%/2 mm and 3%/3 mm acceptance criteria evaluated in PTV1, PTV2, and PTV3 (if available)

	Dataset		
	External (VB20P data)	Local (VE11P data)	
	sCT	MR-AC_Bone_ map	sCT
γ_3%/3mm_ pass rate			
PTV1	99.8 ± 0.3	99.0 ± 0.6	99.6 ± 0.4
	(99.3; 100.0)	(98.2; 100.0)	(98.9; 100.0)
PTV2	99.7 ± 0.2	98.8 ± 2.2	99.7 ± 0.5
	(99.4; 99.9)	(92.9; 100.0)	(98.5; 100.0)
PTV3		98.8 ± 1.2	99.7 ± 0.8
		(96.0; 99.9)	(97.4; 100.0)
γ_2%/2mm_ pass rate			
PTV1	98.9 ± 0.9	95.8 ± 2.7	98.8 ± 0.8
	(97.7; 99.9)	(91.3; 99.7)	(97.3; 99.7)
PTV2	98.1 ± 1.0	95.1 ± 9.7	99.0 ± 0.7
	(97.3; 99.5)	(67.7; 99.8)	(97.5; 99.9)
PTV3		95.1 ± 4.5	98.9 ± 1.2
		(82.8; 99.9)	(95.7; 99.9)

*Abbreviations*: MR-AC = magnetic resonance-based attenuation correction; PTV = planning target volume; PTV1 = primary planning target volume; PTV2 = the high risk of subclinical spread; PTV3 = low risk of subclinical spread; sCT = synthetic computed tomography.

### Local cohort

The ME, MAE, and the dice coefficients of the sCT for the local cohort are also shown in Table 2, where the results and trends are similar to those of the external cohort. Table 2 further shows the results for the vendor-provided MR-AC_Bone_ maps, where the errors for all the compartments are higher and the dice coefficients for bone are correspondingly lower relative to sCT.

The sCT of a representative patient from the local cohort is shown in Figure 1B alongside the input MRIs and the corresponding reference CT. It is apparent that the MRIs have a higher image resolution after the upgrade, which can be seen when compared to Figure 1A. However, the relative image contrast between different tissues is similar before and after upgrade, as the acquisition relies on the same type of sequence. It can also be noticed that the MRI has a larger axial FOV as it is composed of 2 bed positions. The sCT resembles the reference CT and is more detailed compared to the sCT in Figure 1A, due to the improved MRI resolution.

The systematic one-by-one visual inspection of each sCT/CT pair and the corresponding Dixon MRI revealed frequent MRI signal voids due to metallic dental implants, which may affect the sCT quality. Approximately half of the patients from the local cohort had dental implants clearly visible on MRI manifesting as signal voids that varied greatly in size and shape. For a few patients, the artifacts were rather small (∼1 cm) and showed no impact on the sCT, whereas most were larger (∼2 cm) and the model's ability to handle these artifacts varied (Fig. 2B-C). For a single case the artifact was deemed so critical for the sCT that it was excluded from the dosimetric analyses (Fig. 2D). Two additional patients from this cohort were also excluded due to the use of mouthpieces not visible on MRI.

The results of the gamma analyses (Table 3) show high pass rates for the sCT with an average above 98.8% regardless of the acceptance criteria and volume. The corresponding pass rates are lower for MR-AC_Bone_ maps with a single worst γ_2%/2mm_ pass rate of 67.7%, which can be attributed to incorrect tissue segmentations (Fig. E1). Differences in DVH points between the CT-based and sCT-based dose calculations are seen in Figure 3E and 3F. For PTV1, PTV2 and PTV3 relative differences are within ± 2% and in 95% of cases within ± 1% (Fig. 3E). The absolute dose differences in DVH points of the OARs are within ± 1.1 Gy and most (87% of cases) within ± 0.5 Gy (Fig. 3F). Similar to the external cohort, no DVH points for any of the OARs differ by more than 2%, when including volumes with a D_max_ above 10 Gy. Figure 3G and 3H show DVH curves for 2 patients of the local cohort.

## Discussion

In this study we explored the robustness of a previously developed deep learning model for generating sCT from MRI when applying it to a completely independent dataset from another hospital. As the model relies only on images from the Dixon MRI sequence, which is routinely performed for AC purposes on all Siemens Biograph mMR PET/MRI systems, it was therefore directly applicable to a retrospective dataset from the external site. The model demonstrated results that were similar to those of our previous study from which the model originally arises.^10^ Specifically, we obtained similar results for soft tissue (MAE of 48 ± 3 HU vs 41 ± 4 HU) and bone (MAE of 257 ± 45 HU vs 258 ± 51 HU) compartments, whereas we have achieved improved results for air (MAE of 117 ± 12 HU vs 300 ± 69 HU), which could be due to differences in the evaluations and the FOVs. The fact that the model performance is just as good in an independent external test as in the leave-one-out validation on a local dataset performed in the original study, underlines the robustness of the derived model. The model performance is further in accordance with other studies also reporting underestimated bone values, which is to be expected given the blurred appearance of the sCT and can be attributed to imperfect alignment between CT and MRI training data.^19^^,^^20^ Another contributing factor is that all CT voxels above 250 HU were assumed to be bone despite also including very high HU values originating from metallic implants. Dinkla et al^19^ used a cross validation to report a body MAE of 75 ± 9 HU (compared with our 78 ± 13 HU), while Klages et al^20^ obtained MAEs of 94 ± 10 HU and 103 ± 15 HU for 2 different deep leaning models applied to a test dataset. Also, the dice coefficients we report for bone (range, 0.62-0.73) and air/lungs (range, 0.89-0.93) match those reported by Dinkla et al (bone range, 0.52-0.84; air range, 0.63-0.91).^19^ More importantly, the dosimetric evaluation shows excellent agreement between sCT-based and CT-based dose distributions as almost all differences in targets are within ± 1% and after inspection of the single case exceeding −2% the difference was attributed to misalignment between the sCT and CT in the lower neck region. Other studies have similarly reported less than 2% difference in DVH points.^10^^,^^14^^,^^20^ Another study demonstrated gamma map pass rates (γ_3%/3mm_ of 98.7% ± 1.4% and γ_2%/2mm_ of 95.6% ± 2.9%)^19^ lower than ours, but a direct comparison is difficult due to differences in the analyzed volumes. We report pass rates only within the high dose PTVs in contrast to within a 10% dose threshold, which also includes lower doses.

In this study, we further updated our deep learning model to accommodate changes to input MRIs when acquired with the vendor-provided Dixon AC sequence after a major scanner software upgrade (VB20P to VE11P). After the upgrade the MRIs differed specifically by having an improved image resolution and were furthermore acquired over a larger axial FOV. We used transfer learning from the original model to create an updated model, which was evaluated by a leave-one-out cross validation. These sCT images had ME, MAE, and dice coefficients comparable with the literature as well as the external cohort and exceeded the performance of the MR-AC_Bone_ maps. The errors in the MR-AC_Bone_ maps were primarily attributed to the lack of a complete bone representation, misplacements of the registered bones, and inaccurate segmentation of air compartments. These should therefore be carefully inspected if used for clinical dose calculations. The updated deep learning model exploited the improved image resolution of the MRIs to provide sharper sCT images, which may be important for online registration on the treatment linac. In addition, the updated model was adapted to accommodate inputs of a larger axial FOV extending from the skull to approximately mid lungs (2 bed-positions), which allows for accurate treatment of inferior nodes. The dosimetric evaluation indicated that the updated model could be used clinically with all dose differences in target volumes within ± 2%. These low errors were achieved despite the fact that we report dose-to-medium, which is more sensitive to tissue inhomogeneities instead of dose-to-water from more established algorithms like AAA.^31^

Although no explicit test data were available for the updated model, the leave-one-out validation simulated a test scenario as no hyperparameters were optimized for improving model performance. Network alterations were kept to a minimum, changing only input size, training duration, and loss function from mean squared error to mean absolute error as this is known to reduce blurring.^27^^,^^28^ Nevertheless, an independent test dataset is still required prior to eventual clinical implementation.

This study uses a deep learning U-net for deriving sCT, which has the clear advantage over atlas-based methods by being computationally faster and more suitable for patients with abnormal anatomical.^11^

However, our deep learning strategy also has some limitations. First, it is only capable of modeling what is reflected in the training database and both models used in this study are trained on small datasets of head and neck patients. However, for both models, transfer learning has been used to maintain model robustness as the original model was created from a model pretrained with >800 head scans. Furthermore, a recent study also addressing the challenges of converting Dixon-MRI to sCT, when the MRI differed after a software upgrade, concluded that just 5 patients were needed for updating a model to provide a clinically acceptable performance.^21^ Second, the blurred appearance of the sCT is partly caused by imperfect alignment between the MRI and CT pairs in the training data and the lower resolution of the MRI compared to the CT. Although still slightly blurred, the updated model showed more detail in the sCT partly due to the improved image resolution and potentially also because of the choice of loss function. Third, the quality of the sCT is sensitive to artifacts in underlying input images. This is problematic for patients with metal implants significantly affecting the MRI signal, and although we have demonstrated that the model to some extend is capable of handling such artifacts (Fig. 2A-C), it was not able to fully correct for severe artifacts and we had to exclude one such patient in the dosimetric evaluation (Fig. 2D). However, as it was demonstrated in another study, a larger training cohort exposing the network to an increasing number of similar artifacts will improve model robustness.^21^ If the improvements are not completely satisfactory manual corrections could be applied, as is already the case for streaking artifacts in CT and for some cases the sCT may even provide a better alternative (Fig. 2A).

The proposed models for sCT generation could be susceptible to other types of artifacts besides metal artifacts (eg, motion). Future work should focus on improving the model robustness towards region-specific challenges by using a larger and more diverse training cohort. Furthermore, prior to clinical implementation, we suggest a quality assurance strategy for detecting model-specific failures by comparing the output to another and independent algorithm (eg, an atlas-based method) for generating sCT.

In this study we also excluded 2 patients for whom radiotherapy mouthpieces were used, as these devices are not visible on MRI and therefore was not translated into the sCT. Besides trying to make the model infer such devices by training on a larger dataset, another potential solution could be to include MRI visible landmarks on the device and subsequently add a CT-based template manually.

Besides the challenge of accurate dose calculation, radiotherapy planning using solely PET/MRI is challenging because of other important factors such as reliable patient positioning (ensured in this study); the ability of the sCT to match with cone beam CT as a means to determine couch movement on the linac; geometric distortions for MRI; and finally, attenuation correction of PET. These challenges were addressed in our previous study.^10^

## Conclusions

We have studied the robustness and generalizability of a previously developed deep learning method for deriving sCT from MRI for radiotherapy usage. The model was applied to a completely independent external dataset and was furthermore updated to accommodate scanner software-induced MRI changes and a larger axial FOV extending from the skull to mid thorax. In both cases, the derived sCT images produced radiotherapy dose distributions that were very similar to those calculated on the reference CT suggesting a robust, generalizable, and reliable method.

## Acknowledgements

We thank the patients in both Rigshospitalet and Guy's and St. Thomas’ NHS Foundation Trust. We thank John and Birthe Meyer Foundation, Denmark for donating the PET/MRI system to Rigshospitalet. We thank IBM Denmark for providing 2 POWER9 servers with 4 Tesla V100 GPUs in each system.

## Appendix Supplementary materials

Image, application 1

## Footnotes

Supplementary material associated with this article can be found in the online version at doi:10.1016/j.adro.2021.100762.
