# Supplementary material for: Robustness and Generalizability of Deep Learning Synthetic Computed Tomography for Positron Emission Tomography/Magnetic Resonance Imaging–Based Radiation Therapy Planning of Patients With Head and Neck Cancer
Source: Adv Radiat Oncol. 2021 Jul 26;6(6):100762. doi: 10.1016/j.adro.2021.100762 (PMC8452789; doi:10.1016/j.adro.2021.100762)
Supplement: Supplementary file 1 [file mmc1.docx]

# SUPPLEMENTARY


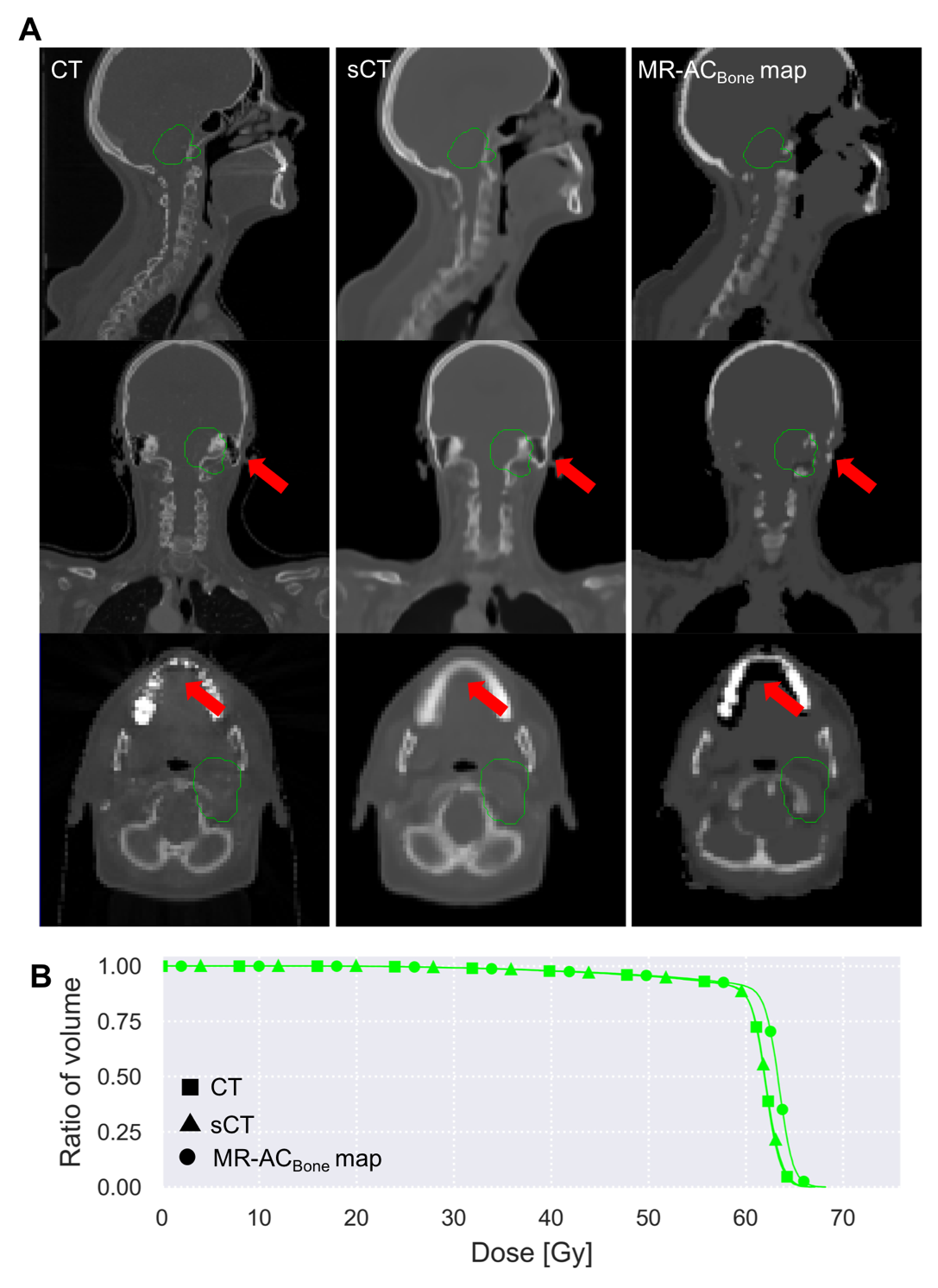


Figure E1: (A) Patient example showing the CT, sCT, and MR-AC_Bone_ map. Red arrows indicate areas where the MR-AC_Bone_ map has incorrect tissue classification which translates into the dose distribution yielding a γ_2%/2mm_ pass rate of 67.7% for the PTV2 target volume shown in green. (B) Shows the DVH curve for the target volume for the CT, sCT, and MR-AC_Bone_ map.
